# TAIII Suppresses the Growth of T790M-Mutant Non-Small-Cell Lung Cancer by Targeting the EGFR/ERK Signaling Pathway

**DOI:** 10.3390/ph18101431

**Published:** 2025-09-24

**Authors:** Shang Gao, Ying Luan, Xinhao Yu, Ludan Wang, Xuefeng Huang, Jian Yang, Wei Liu

**Affiliations:** 1 Department of Pharmacology, Nanjing Medical University, Nanjing 211166, China; 15651885632@163.com (S.G.); luanying@stu.njmu.edu.cn (Y.L.); 15151474924@163.com (X.Y.); wld@stu.njmu.edu.cn (L.W.); 2 Department of Natural Medicinal Chemistry, School of Chinese Pharmacy, China Pharmaceutical University, Nanjing 210009, China; hxf@cpu.edu.cn

**Keywords:** TAIII, NSCLC, EGFR T790M, ERK signaling pathway, autophagy–lysosomal pathway, drug interaction

## Abstract

**Background/Objectives**: First-generation EGFR tyrosine kinase inhibitors (EGFR-TKIs) are standard first-line treatments for advanced non-small-cell lung cancer (NSCLC). However, acquired resistance often develops via secondary T790M mutations, necessitating new therapies. Timosaponin AIII (TAIII) shows antitumor activity and has been found to suppress EGFR phosphorylation. This study aimed to evaluate the therapeutic potential of TAIII in overcoming T790M-mediated resistance in NSCLC and elucidate its underlying molecular mechanisms. **Methods**: We evaluated the inhibitory effects of TAIII on proliferation (EdU assay) and migration (Transwell assay) in T790M-mutated H1975 cells. EGFR phosphorylation and downstream signaling were analyzed by Western blotting. Molecular docking was employed to predict the binding of TAIII to EGFR, while CETSA (cellular thermal shift assay) and SIP (Stability of Interaction Partners) assays were used to validate TAIII-EGFR interaction stability. The in vivo antitumor efficacy was further confirmed in nude mouse xenograft models. **Results**: TAIII inhibited H1975 cell proliferation and migration by downregulating p-EGFR (Y1068) and ERK signaling. Docking showed stable TAIII binding in the EGFR kinase domain via hydrogen bonds at THR-776 and PRO-770, confirmed by CETSA and SIP. At high concentrations, TAIII induced EGFR degradation through autophagy–lysosome pathways. TAIII monotherapy outperformed combinations with gefitinib (CI > 1). Xenograft models confirmed its potent antitumor effect via EGFR phosphorylation inhibition. **Conclusions**: TAIII demonstrates substantial therapeutic potential for overcoming T790M-mediated resistance in NSCLC by its dual mechanism of EGFR signaling inhibition and receptor degradation, supporting further preclinical and clinical development.

## 1. Introduction

Lung cancer remains a significant global health burden, with China experiencing a concerning rise in both incidence and mortality rates in recent years [1]. The 2020 Global Cancer Statistics report revealed China accounted for approximately 820,000 new lung cancer cases and 715,000 lung cancer-associated deaths, highlighting its substantial disease burden [2]. Non-small-cell lung cancer (NSCLC), constituting 80–85% of all lung cancer cases, represents a leading cause of cancer-related mortality worldwide [3]. Current therapeutic modalities for NSCLC include surgical resection [4], systemic chemotherapy [5], radiotherapy [6], molecularly targeted agents [7], and immunotherapy [8]. While molecular targeted therapies have demonstrated improved clinical outcomes in recent years, significant challenges remain [9]. The development of drug resistance—mediated through secondary mutations [10] or bypass pathway activation [11]—continues to limit their long-term efficacy. These persistent therapeutic limitations underscore the critical need for novel pharmacological interventions.

The epidermal growth factor receptor (EGFR), a critical receptor tyrosine kinase [12], plays a pivotal role in lung cancer pathogenesis and progression through its mutational activation [13]. Ligand binding (e.g., EGF) triggers EGFR dimerization and subsequent activation of downstream signaling cascades, including the PI3K/AKT [14], RAS/RAF/MAPK [15], and JAK/STAT3 [16] pathways, which play important roles in cell proliferation, survival and migration. To target EGFR-driven oncogenesis, multiple generations of EGFR tyrosine kinase inhibitors (TKIs) have been developed, including first-generation (e.g., gefitinib) [17], and second-generation (e.g., afatinib) [18] agents. These small-molecule inhibitors exert their therapeutic effects by competitively binding the ATP pocket of EGFR kinase domain, thereby suppressing oncogenic signaling [19]. Despite initial clinical efficacy, EGFR-TKI treatment inevitably leads to acquired resistance, typically emerging within 8-18 months [20]. The resistance mechanisms include the T790M mutation [21], activation of EGFR downstream signaling molecules [22], bypass activation [23], and phenotypic transformation [24]. Among these, the T790M gatekeeper mutation represents the most prevalent mechanism of acquired resistance [25].

The T790M mutation, a critical gatekeeper residue within the EGFR ATP-binding pocket, confers enhanced ATP-binding affinity that compromises the efficacy of competitive ATP inhibitors [25]. This increased affinity reduces the binding potential of first-generation tyrosine kinase inhibitors (TKIs; e.g., gefitinib), thereby diminishing their therapeutic effect. Additionally, the T790M mutation may potentiate EGFR kinase activity, resulting in constitutive ERK pathway activation through sustained phosphorylation [26]. Clinically, this mutation emerges in 50–60% of EGFR-TKI-resistant cases [27]. Although combination strategies (e.g., EGFR-TKIs with chemotherapy/immunotherapy) demonstrate improved therapeutic outcomes, they concurrently increase adverse events, including rash, diarrhea, and hepatic dysfunction [28]. These adverse effects may compromise patients’ quality of life and treatment compliance [26]. Consequently, the development of novel therapeutic agents and strategies that effectively target T790M-mediated resistance while maintaining favorable safety profiles represents a critical unmet need in precision oncology.

Natural compounds have garnered significant attention in cancer therapy due to their favorable safety profiles and cost-effectiveness [29]. Among these, *Anemarrhena asphodeloides* Bunge, a well-established medicinal plant in traditional Chinese medicine, has been widely recognized for its diverse therapeutic properties including antipyretic, detoxifying, antidiarrheal, and sedative effects [30]. Timosaponin AIII (TAIII), a bioactive steroidal saponin isolated from this plant, has attracted considerable research interest due to its multifaceted pharmacological activities [31]. Current evidence demonstrates that TAIII exhibits a broad spectrum of biological effects, ranging from anti-inflammatory, antipyretic, and anticoagulant properties to significant antitumor activity against multiple cancer types [32].

Emerging studies have begun elucidating the therapeutic potential and molecular mechanisms of TAIII in NSCLC. Current evidence indicates that TAIII exerts potent antitumor effects through multiple programmed cell death pathways, including apoptosis [33], autophagy [34], and ferroptosis [35]. However, its efficacy against EGFR-TKI-resistant NSCLCs, particularly those harboring T790M mutations, remains unexplored. Our recent work has revealed that TAIII significantly downregulates EGFR phosphorylation while upregulating drug-metabolizing enzymes [36]. These findings support the hypothesis that TAIII may function as a novel EGFR-targeting agent, potentially offering therapeutic benefits for both treatment-naive and T790M-mutant NSCLC cases.

In this study, we systematically evaluated the antitumor activity and molecular mechanisms of TAIII against T790M-mutant NSCLC through in vitro and in vivo studies. Our findings elucidate the molecular mechanisms by which TAIII overcomes acquired resistance mediated by the T790M epidermal EGFR mutation in NSCLC, which will facilitate the development of novel therapeutic strategies for drug-resistant NSCLC.

## 2. Results

### 2.1. TAIII Suppresses the Viability, Proliferation, and Migration of T790M-Mutant H1975 Cells

**Figure 1:**
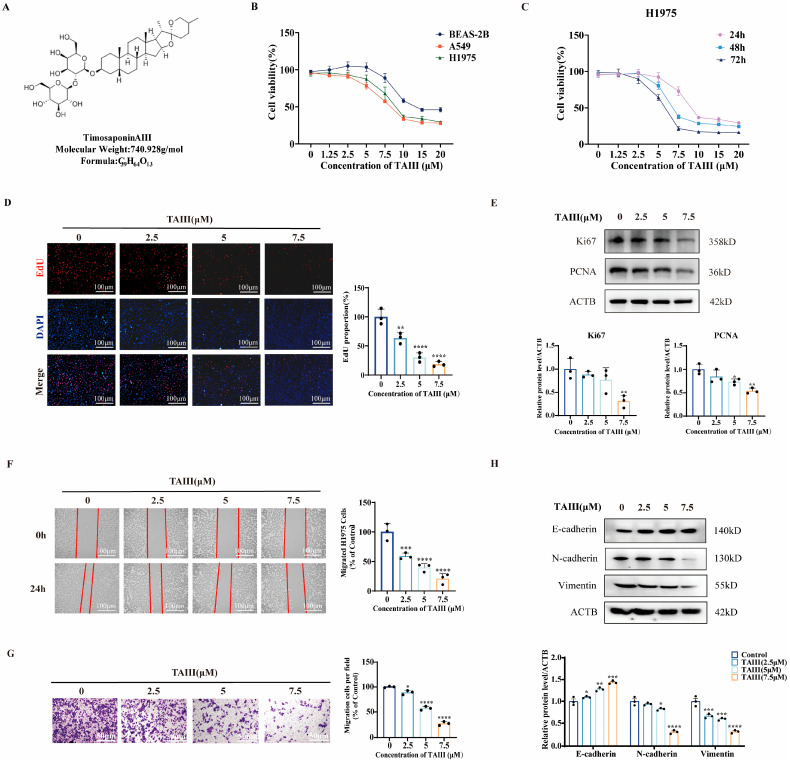
Effects of TAIII on the viability, proliferation, and migration of T790M-mutant H1975 cells. (**A**) Chemical structure of TAIII. (**B**) Effects of TAIII on cell viability across three cell lines, as determined by CCK-8 assay. (**C**) Time-dependent inhibition of H1975 cell viability assessed by CCK-8 assay at 24, 48, and 72 h post-treatment. (**D**) Anti-proliferative effects of TAIII (0, 2.5, 5, and 7.5 μM) on H1975 cells after 24 h of treatment, as measured by EdU assay. (**E**) Western blotting analysis of proliferation-associated markers (Ki67 and PCNA) following TAIII (0, 2.5, 5, and 7.5 μM) treatment for 24 h. (**F**) Wound healing assay and (**G**) Transwell assay were used to evaluate the migratory capacity of H1975 cells treated with TAIII (0, 2.5, 5, and 7.5 μM) for 24 h. (**H**) Western blot analysis of epithelial–mesenchymal transition (EMT)-related markers (E-cadherin, N-cadherin, and vimentin) following 24 h treatment with TAIII (0, 2.5, 5, and 7.5 μM). The data are presented as the mean ± SD (*n* = 3). Statistical significance was analyzed by one-way ANOVA. The symbols used to indicate significance levels are as follows: * *p* < 0.05, ** *p* < 0.01, *** *p* < 0.001, **** *p* < 0.0001 vs. control.

To rigorously evaluate the anti-T790M-mutant NSCLC activity of Timosaponin AIII, we performed cell viability analyses using the CCK-8 assay in H1975 cells (EGFR T790M mutant cells). Meanwhile, we detected cell viabilities of BEAS-2B cells (normal human bronchial epithelial cells) and A549 cells (EGFR WT cells) after TAIII treatment. The results showed that TAIII significantly inhibited, in a concentration-dependent manner, H1975 cells’ viability, with an IC50 value of 8.560 μM (Figure 1B). The cell viabilities of BEAS-2B cells and A549 cells decreased, with IC50 values of 15.510 and 8.678 μM, respectively. We further characterized the temporal dynamics in H1975 cells. As shown in Figure 1C, the inhibitory effect of TAIII on H1975 cells occurred in a time-dependent manner.

To further explore the effect of TAIII on the proliferation of H1975 cells, we performed an EdU staining assay. As illustrated in Figure 1D, H1975 cell proliferation exhibited a concentration-dependent suppression upon treatment with escalating concentrations of TAIII (0, 2.5, 5, and 7.5 μM). Additionally, we analyzed the expression of key proliferation markers, Ki67 and PCNA, via Western blotting. Consistent with the EdU assay results, Figure 1E demonstrated a marked concentration-dependent reduction in both Ki67 and PCNA protein levels following TAIII treatment. These data provide compelling evidence that TAIII effectively inhibits the proliferation of H1975 cells.

To further investigate the influence of TAIII on the migratory potential of H1975 cells, we employed both wound healing and Transwell assays. As demonstrated in Figure 1F, TAIII treatment resulted in a concentration-dependent inhibition of wound closure in the scratch assay. This finding was further corroborated by the Transwell assay (Figure 1G). To elucidate the potential mechanism underlying this antimigratory effect, we analyzed the expression of key epithelial–mesenchymal transition (EMT) markers using Western blotting (Figure 1H). Notably, TAIII treatment led to a significant upregulation of E-cadherin (an epithelial marker). Conversely, the expression of mesenchymal markers, including N-cadherin and vimentin, was markedly downregulated with increasing concentrations of TAIII. These results demonstrated that TAIII effectively suppresses the migratory capacity of H1975 cells, potentially through modulation of EMT-related pathways.

Collectively, these findings demonstrate that TAIII effectively inhibits the viability, proliferation, and migration of T790M-mutant H1975 cells, suggesting its potential as a therapeutic agent against T790M-mediated resistance.

### 2.2. TAIII Potently Suppresses EGFR Phosphorylation and Its Downstream Signaling Cascades in T790M-Mutant H1975 Cells

**Figure 2:**
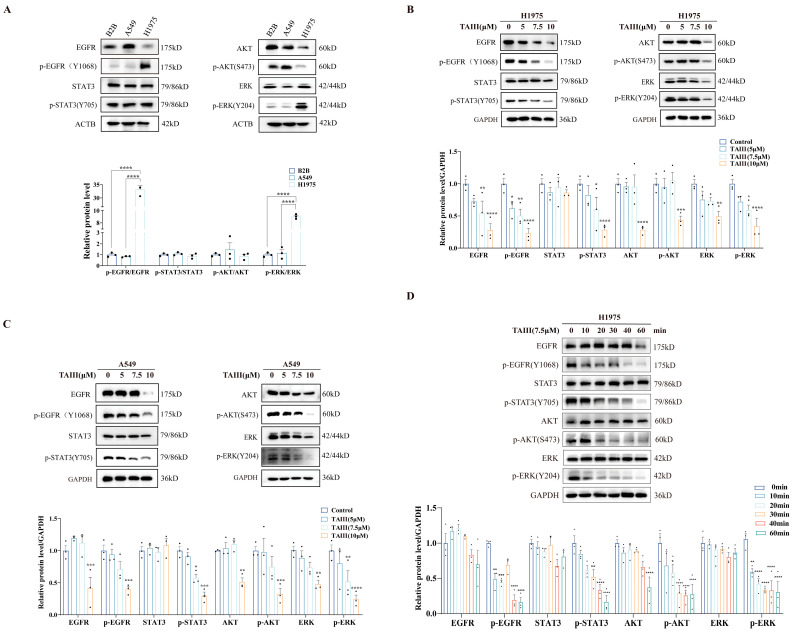
Effects of TAIII on the EGFR phosphorylation and its downstream signaling cascades in T790M-mutant H1975 cells. (**A**) Western blotting analysis was used to detect the basal activation status of EGFR and downstream effectors (AKT, ERK, STAT3) in BEAS-2B, A549, and H1975 cells. (**B**) Western blotting analysis of activation status of EGFR and downstream effectors in H1975 cells after TAIII (0, 5, 7.5, and 10 μM) treatment for 24 h. (**C**) Western blotting analysis of activation status of EGFR and downstream effectors in A549 cells after TAIII (0, 5, 7.5, and 10 μM) treatment for 24 h. (**D**) Western blotting analysis of activation status of EGFR and downstream effectors in H1975 cells after TAIII (7.5 μM) treatment over 60 min. The data are presented as the mean ± SD (*n* = 3). Statistical significance was determined via one-way ANOVA (**A**–**C**) and one-way repeated measures ANOVA (**D**). The symbols used to indicate significance levels are as follows: * *p* < 0.05, ** *p* < 0.01, *** *p* < 0.001, **** *p* < 0.0001 for (**B**–**D**) vs. control.

Next, Western blotting analysis was performed to assess the basal expression profiles of EGFR and its downstream signaling effectors in BEAS-2B, A549, and H1975 cell lines (Figure 2A). Notably, H1975 cells exhibited markedly elevated phosphorylation levels of EGFR (Y1068) and ERK (Y204) compared with BEAS-2B and A549 cells. This hyperactivation phenotype likely stems from the T790M mutation-induced conformational change in EGFR, which enhances its ATP-binding affinity and, consequently, leads to sustained downstream pathway activation. These results provide compelling evidence that the EGFR-ERK signaling axis is constitutively activated in H1975 (EGFR T790M) cells, thereby establishing this pathway as a potential therapeutic target for TAIII intervention. To investigate the effects of TAIII on EGFR and its downstream signaling cascades, H1975 cells were treated with escalating concentrations (0, 5, 7.5, and 10 μM) for 24 h, followed by comprehensive signaling pathway analysis (Figure 2B). Dose-dependent reductions were observed in the phosphorylation of EGFR (Y1068), AKT (S473), and ERK (Y204) following TAIII treatment. Notably, at the highest concentration tested (10 μM), the total protein expressions of EGFR, AKT, and ERK were significantly downregulated, while STAT3 levels remained unaltered. These results demonstrate that TAIII not only attenuates EGFR-mediated signaling through the inhibition of receptor and downstream effector phosphorylation but also reduces total protein expression of key signaling molecules at higher concentrations. To confirm the mechanism of TAIII on the EGFR signaling pathway, we extended our investigation to A549 cells (Figure 2C). Western blotting analysis revealed that TAIII treatment similarly attenuated phosphorylation of EGFR and its downstream effectors (AKT and ERK) in A549 cells. Notably, high-concentration treatment (10 μM) also reduced the total protein expression of EGFR, AKT, and ERK in A549 cells, consistent with the pattern seen in H1975 cells.

To delineate the temporal dynamics of TAIII-mediated EGFR pathway inhibition in T790M-resistant H1975 cells, we performed a kinetic study using 7.5 μM treatment over a 60 min timeframe (0, 10, 20, 30, 40, and 60 min; Figure 2D). The results showed that significant attenuation of EGFR autophosphorylation at Y1068 and inhibition of ERK activation (Y204 phosphorylation) were observed after TAIII treatment for 10 min. Meanwhile, suppression of AKT phosphorylation (S473) and reduction in STAT3 activation (Y705 phosphorylation) were detected by 20 and 30 min, respectively. Notably, total protein levels of EGFR, STAT3, AKT, and ERK remained stable throughout the 60 min observation period, indicating that the acute effects of TAIII are mediated through phosphorylation inhibition rather than protein degradation. The observed temporal pattern of pathway inhibition strongly supports EGFR as the primary molecular target through which TAIII exerts its anti-T790M-resistant NSCLC activity.

### 2.3. TAIII Acts as an EGFR Inhibitor

**Figure 3:**
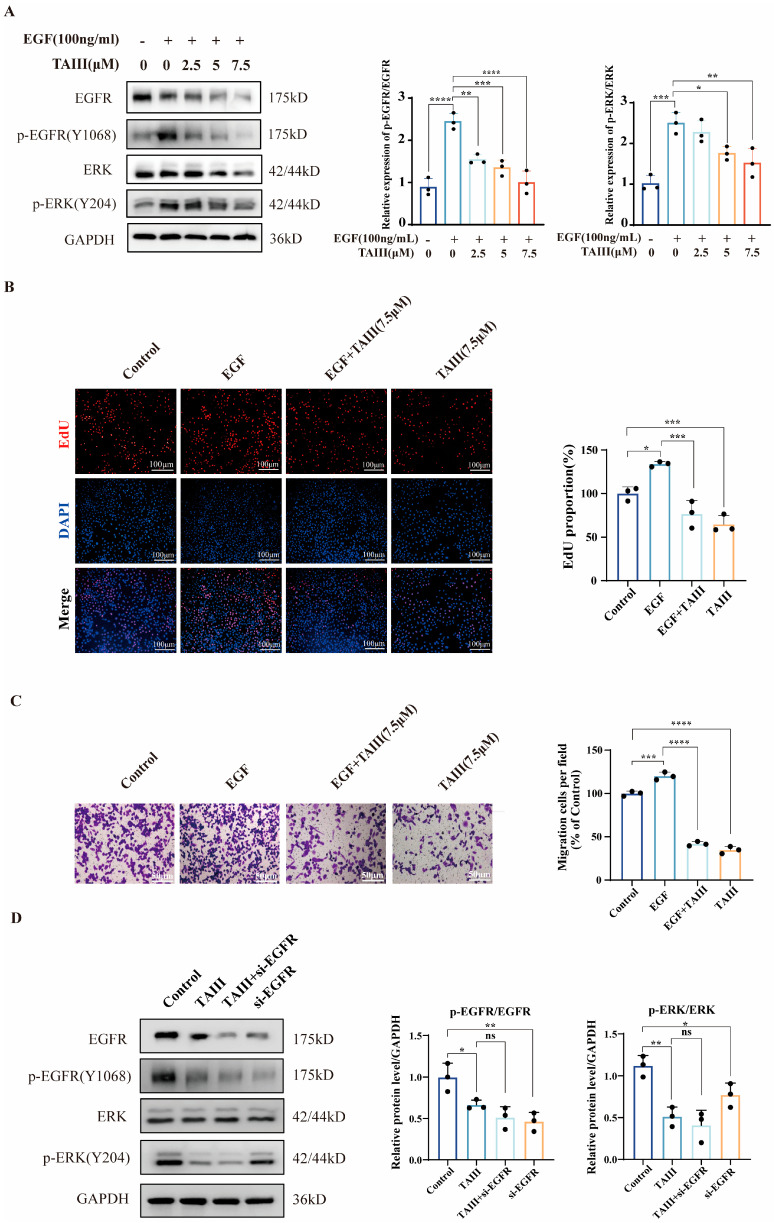
TAIII acts as an EGFR inhibitor. H1975 cells were stimulated with EGF (50 ng/mL, 30 min) followed by treatment with varying concentrations of TAIII (0, 2.5, 5, and 7.5 μM) for 24 h. (**A**) Western blotting was performed to assess the expression levels of EGFR, p-EGFR (Y1068), ERK, and p-ERK (Y204). (**B**) The effect on cell proliferation was evaluated using the EdU assay. (**C**) Cell migration was subsequently analyzed via Transwell assay. (**D**) Cells were transfected with EGFR-targeting siRNA for 48 h to knock down EGFR expression. Western blotting was then conducted to measure changes in EGFR, p-EGFR (Y1068), ERK, and p-ERK (Y204). The data are presented as the means ± SD (*n* = 3). Statistical analysis was performed using one-way ANOVA. The symbols used to indicate significance levels are as follows: * *p* < 0.05, ** *p* < 0.01, *** *p* < 0.001, **** *p* < 0.0001, and ns for *p* > 0.05.

To establish direct EGFR kinase inhibition by TAIII, we employed a ligand-competition experiment. This approach evaluated the compound’s ability to antagonize EGF-mediated receptor activation and subsequent downstream signaling cascade initiation. H1975 cells were pretreated with TAIII (0, 2.5, 5, or 7.5 μM) for 24 h, followed by acute exposure to EGF (100 ng/mL) for 30 min. As shown in Figure 3A, EGF stimulation markedly increased p-EGFR (Y1068) levels, whereas TAIII treatment attenuated this effect. A similar expression pattern was observed for p-ERK (Y204), suggesting that TAIII effectively suppresses EGF-induced downstream signaling. These findings imply that TAIII may function as an EGFR inhibitor, a hypothesis further supported by subsequent pharmacodynamic assays. In Figure 3B, EdU proliferation assays revealed that EGF (100 ng/mL) significantly enhanced H1975 cell proliferation, an effect that was substantially mitigated by TAIII. Similarly, Transwell migration assays (Figure 3C) demonstrated that EGF-induced migratory capacity was markedly reduced upon TAIII treatment. Together, these results reinforce the conclusion that TAIII exerts anti-resistance effects by targeting EGFR.

To further validate EGFR as the molecular target of TAIII, we performed EGFR knockdown using small interfering RNA (si-EGFR, 30 nM) and analyzed p-EGFR (Y1068) and p-ERK (Y204) expression via Western blotting. As depicted in Figure 3D, EGFR silencing significantly reduced the phosphorylation levels of both EGFR and ERK, paralleling the effects of 7.5 μM TAIII alone. Notably, combined treatment with TAIII and si-EGFR did not further enhance the suppression of p-EGFR or p-ERK beyond that achieved by either intervention independently. These data collectively demonstrate that EGFR is a critical target of TAIII inhibitory effects on the proliferation and migration of H1975 (EGFR T790M) cells.

### 2.4. Molecular Interaction and Binding Stability of TAIII with EGFR

**Figure 4:**
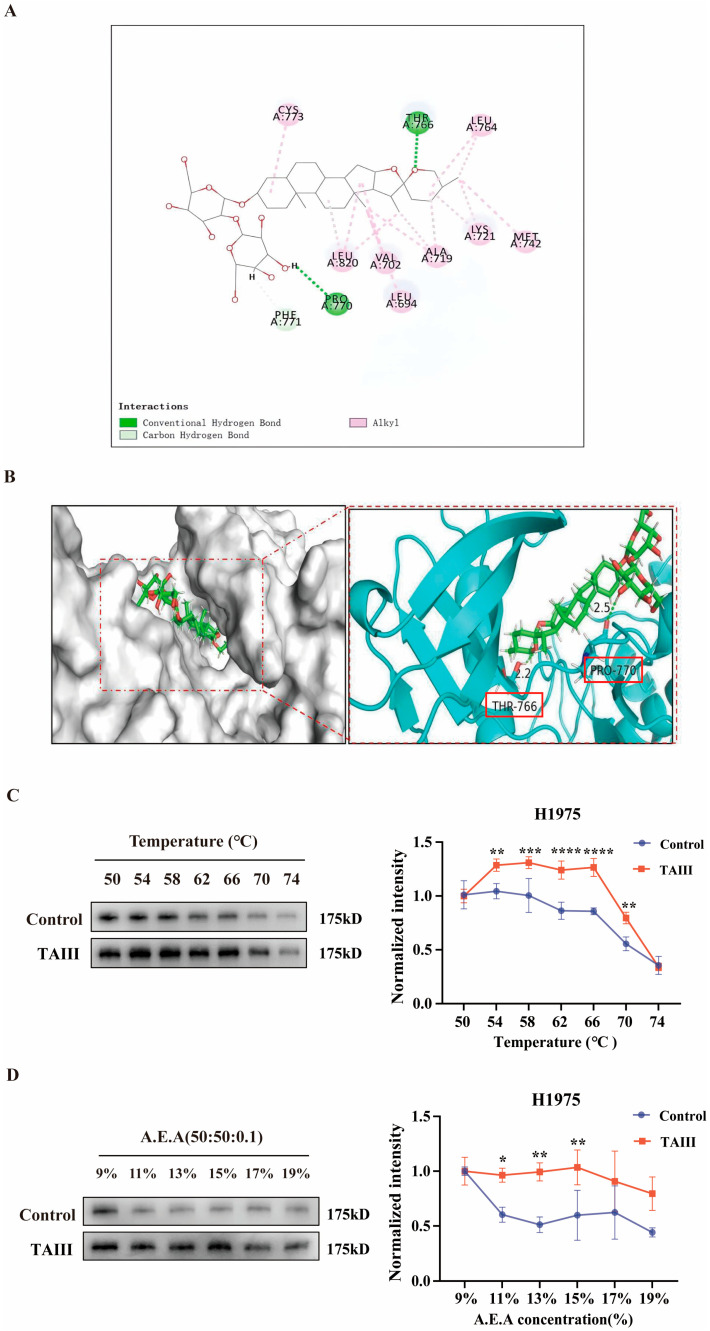
Molecular interaction and binding stability of TAIII with EGFR. (**A**) Two-dimensional schematic representation of TAIII–EGFR binding interactions. (**B**) Three-dimensional structural model of TAIII docked in the EGFR binding pocket. (**C**) Thermal stability profiling of the TAIII–EGFR complex using CETSA following treatment with 20 μM TAIII. (**D**) The stability of TAIII binding to the EGFR was detected using SIP assay following treatment with 20 μM TAIII. Data are expressed as mean ± SD (*n* = 3). Statistical analysis was performed using one-way repeated measures ANOVA. The symbols used to indicate significance levels are as follows: * *p* < 0.05, ** *p* < 0.01, *** *p* < 0.001, **** *p* < 0.0001.

To elucidate the molecular interactions between TAIII and EGFR, we performed molecular docking studies using PyMOL software (version 3.1.6.1) Figure 4A,B). Structural analysis revealed that TAIII binds tightly within the pocket of EGFR, with its steroidal saponin moiety forming a key hydrogen bond with THR-776. The glycosyl group extends toward the protein’s hydrophilic surface, where the terminal hydroxyl group establishes an additional hydrogen bond with PRO-770. These dual hydrogen-bonding interactions at opposite ends of the molecule effectively anchor the ligand in EGFR’s active site, potentially explaining its potent biological activity. These structural findings suggest that TAIII exerts its antitumor effects through direct EGFR inhibition.

To further validate the TAIII–EGFR interaction, we employed CETSA and SIP assays. CETSA monitors ligand-induced changes in protein thermal stability, while SIP evaluates binding affinity through chemical denaturation resistance. As shown in Figure 4C,D, both assays demonstrated TAIII could increase stabilization of EGFR. In CETSA experiments, EGFR protein levels showed significantly reduced thermal denaturation (50–74 °C range) in TAIII-treated samples. Similarly, SIP assays revealed enhanced resistance to denaturation by A.E.A. solution (acetone/ethanol/acetic acid = 50:50:0.1; 9–19% concentration range) in the presence of TAIII. These results collectively demonstrated that TAIII binding confers substantial stability to EGFR, further supporting its role as a direct EGFR inhibitor.

### 2.5. Mechanism of EGFR Degradation by High Concentration of TAIII

**Figure 5:**
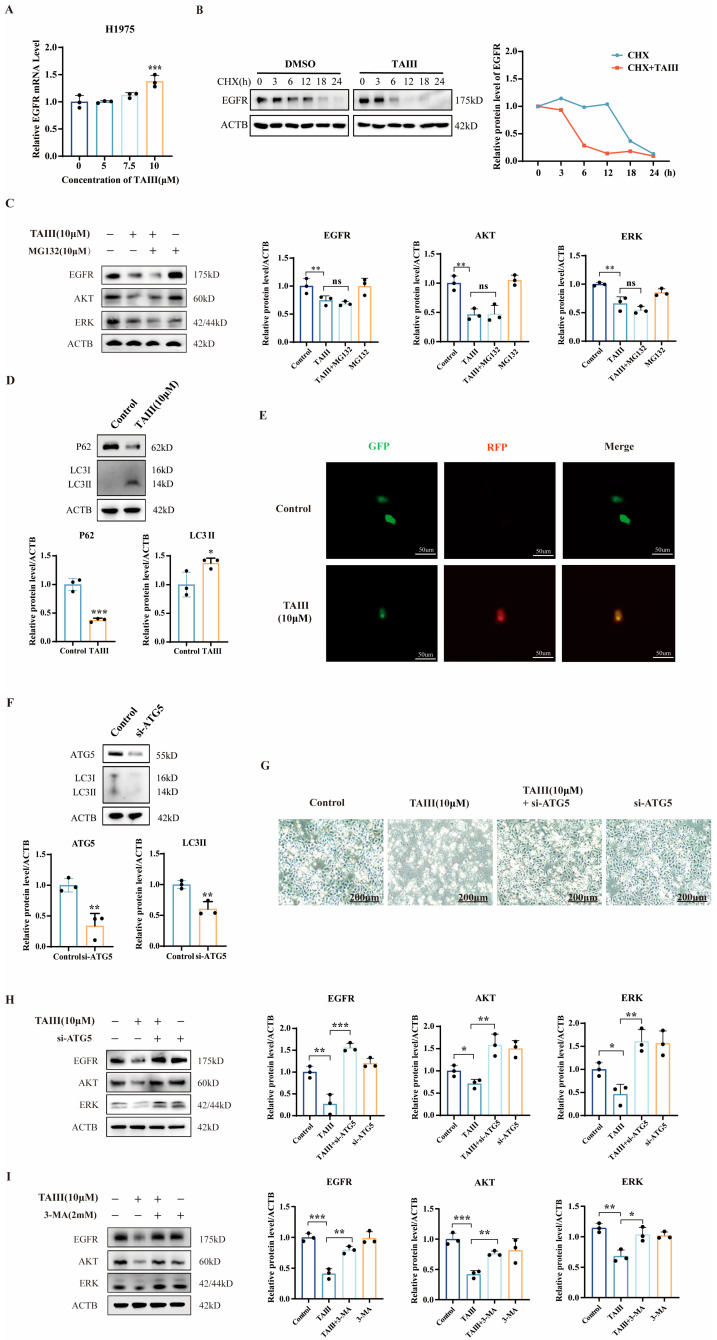
Mechanism of EGFR degradation by TAIII at high concentrations. (**A**) RT-qPCR quantified EGFR mRNA levels in H1975 cells treated with TAIII (0, 5, 7.5, and 10 μM) for 24 h. (**B**) The kinetics of EGFR protein degradation were assessed using a CHX chase assay following treatment with TAIII (10 μM) for 0, 3, 6, 12, 18, and 24 h. (**C**) The effects of the proteasome inhibitor MG-132 on the expressions of EGFR, AKT, and ERK were evaluated. Cells were pretreated with MG-132 for 6 h prior to a 24 h exposure to TAIII. (**D**) Western blotting analysis of the expression of key autophagy-associated proteins (p62 and LC3) upon TAIII (10 μM) treatment for 24 h. (**E**) H1975 cells transfected with pGFP-RFP-LC3 plasmid were treated with 10 μM TAIII for 12 h and observed under an inverted fluorescence microscope (200×). Yellow puncta (resulting from the merged GFP (green) and RFP (red) signals) indicate autophagosomes. (**F**) Western blotting was used to determine the knockdown efficiency of ATG5 and the consequent changes in the autophagy marker (LC3) following 48 h of siRNA transfection. (**G**) Phase-contrast microscopy images (40×) of ATG5-knockdown cells treated with TAIII. (**H**) Western blotting was performed to detect EGFR, AKT, and ERK in ATG5-knockdown H1975 cells treated with TAIII (10 μM) for 24 h. (**I**) The effects of the autophagy inhibitor 3-MA on the expressions of EGFR, AKT, and ERK were evaluated. Cells were pretreated with 3-MA for 1 h prior to a 24 h exposure to TAIII. Data represent mean ± SD (*n* = 3). For comparisons between two independent groups, unpaired *t*-tests were used (**D**,**F**); for other comparisons involving multiple groups, one-way ANOVA was performed. The symbols used to indicate significance levels are as follows: * *p* < 0.05, ** *p* < 0.01, *** *p* < 0.001, and ns for *p* > 0.05.

Given the significant downregulation of EGFR protein expression observed at high concentrations of TAIII (10 μM), we sought to elucidate the underlying molecular mechanism. First, we assessed whether this regulation occurred at the transcriptional level by quantifying EGFR mRNA expression in H1975 cells treated with TAIII (0, 5, 7.5, and 10 μM) using RT-qPCR. Interestingly, as shown in Figure 5A, EGFR mRNA levels were not suppressed, but rather, significantly elevated at 10 μM TAIII, suggesting a potential compensatory feedback mechanism in response to protein depletion and ruling out transcriptional regulation as the primary mode of action. To investigate post-translational regulation, we performed protein stability assays using cycloheximide (CHX) to block new protein synthesis. Western blotting analysis of EGFR protein levels at sequential time points (0, 3, 6, 9, and 12 h) revealed significantly accelerated EGFR degradation in cells treated with 10 μM TAIII compared with controls (Figure 5B). Notably, this enhanced degradation became statistically significant by 6 h post-treatment. These findings collectively demonstrate that TAIII mediates EGFR downregulation by promoting EGFR protein degradation.

There are two major pathways for protein degradation: the ubiquitin–proteasome system and the autophagy–lysosome system. To investigate the pathway by which TAIII induces EGFR protein degradation, we performed the following experiments. First, we used the proteasome inhibitor MG-132 to block the proteasome pathway and examined whether it could reverse the degradation of EGFR. H1975 cells were treated with MG-132 (10 μM) and/or TAIII (10 μM), followed by Western blotting analysis of EGFR, AKT, and ERK protein levels. As shown in Figure 5C, MG-132 did not rescue the TAIII-induced downregulation of EGFR, AKT, and ERK, indicating that TAIII does not promote their degradation via the proteasome pathway. Subsequently, we investigated the effects of TAIII on autophagy in H1975 cells. Western blotting analysis was performed to detect the expression levels of the autophagy-related markers p62 and LC3I/II. The results showed that treatment with TAIII (10 μM) significantly decreased the expression of p62, while markedly increasing the expression of LC3II (Figure 5D). To further monitor autophagic flux dynamics, we employed the GFP-RFP-LC3 dual fluorescent labeling assay. H1975 cells transfected with the pGFP-RFP-LC3 plasmid were treated with 10 μM TAIII for 12 h. Observation under an inverted fluorescence microscope revealed that TAIII-treated cells exhibited yellow fluorescent puncta (formed by the superposition of GFP and RFP signals, indicating autophagosomes), while no significant fluorescent puncta were observed in the control group. These results demonstrate that TAIII treatment effectively induces autophagosome formation (Figure 5E). These findings demonstrate that TAIII induces autophagy in H1975 cells. Subsequently, we specifically manipulated the essential autophagy gene ATG5 (Autophagy Related 5) to regulate autophagy. Using small interfering RNA (si-ATG5) to knock down ATG5 expression, we investigated whether this intervention could reverse the TAIII (10 μM)-induced reduction in EGFR, AKT, and ERK protein levels. The results demonstrated that si-ATG5 significantly reduced ATG5 protein expression, accompanied by decreased LC3-II levels (Figure 5F), confirming successful autophagy inhibition. Western blotting analysis further revealed that ATG5 knockdown markedly reversed the downregulation of EGFR, AKT, and ERK proteins induced by high-concentration TAIII (Figure 5H), providing compelling evidence that TAIII promotes EGFR degradation through the autophagy–lysosomal pathway at higher concentrations. Moreover, phase-contrast microscopy showed that ATG5 silencing significantly attenuated TAIII-induced cell death (Figure 5G), suggesting that autophagy inhibition protects cells by preventing the degradation of EGFR/AKT/ERK signaling components. To further validate these findings, we utilized the autophagy inhibitor 3-methyladenine (3-MA) and found that it similarly restored the protein levels of EGFR, AKT, and ERK—an effect consistent with that observed upon ATG5 knockdown (Figure 5I). Collectively, these findings demonstrate that at higher concentrations, TAIII induces cell death in T790M-mutant H1975 cells by promoting EGFR degradation through the autophagy–lysosomal pathway, subsequently disrupting downstream AKT and ERK signaling.

### 2.6. Combination Treatment of TAIII and Gefitinib

Gefitinib, a first-generation EGFR-TKI, represents the first-line standard therapy for locally advanced or metastatic NSCLC harboring EGFR-sensitive mutations. However, the clinical efficacy of gefitinib is substantially compromised by both primary and acquired resistance mechanisms, which not only reduce treatment response rates but ultimately contribute to disease progression, consequently diminishing patients’ progression-free survival (PFS) and overall survival (OS) [31]. Our previous findings demonstrated that TAIII exhibits potent growth inhibitory activity against H1975 cells (EGFR T790M). Building upon these observations, we sought to evaluate the potential synergistic effects of TAIII when combined with gefitinib in this resistant cell line model.

**Figure 6:**
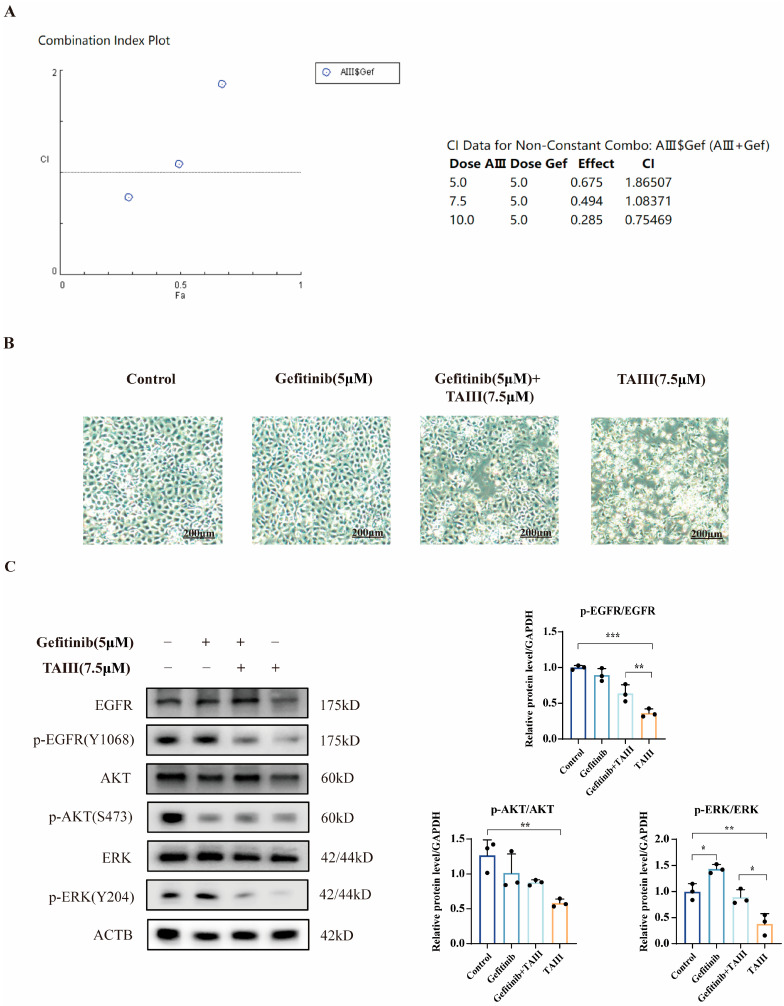
Evaluation of Timosaponin AIII and gefitinib combination therapy. (**A**) Combination index (CI) analysis was performed using CompuSyn software (ComboSyn, PD Science, LLC., Paramus, NJ, USA) to assess drug interactions. (**B**) Morphological changes in cells treated with TAIII and gefitinib combination were examined by phase-contrast microscopy (4× magnification). (**C**) Western blotting analysis of phosphorylation levels of EGFR and downstream signaling proteins following combination treatment with TAIII and gefitinib for 24 h. Data are expressed as mean ± SD (*n* = 3). Statistical analysis was performed using two-way ANOVA, followed by *t*-tests for intergroup comparisons. The symbols used to indicate significance levels are as follows: * *p* < 0.05, ** *p* < 0.01, *** *p* < 0.001.

To quantitatively assess drug interactions, we performed Chou–Talalay combination index (CI) analysis to evaluate the potential synergy between TAIII and gefitinib. The CI, derived from dose-effect curve analysis using the Chou–Talalay method, provides a quantitative measure of drug interactions: CI < 1 indicates synergy (combination effect exceeds individual drug effects), CI = 1 indicates additivity (combination effect equals the sum of individual effects), and CI > 1 indicates antagonism (combination effect is subadditive). Using TAIII concentrations of 5, 7.5, and 10 μM in combination with 5 μM gefitinib, our analysis revealed that at 5 and 7.5 μM TAIII, CI values were 1.865 and 1.084, respectively, indicating antagonistic effects (Figure 6A). Notably, at 10 μM TAIII, we observed synergistic activity (CI = 0.755). Morphological assessment by inverted microscopy at 24 h post-treatment provided further insight (Figure 6B). While 5 μM gefitinib alone showed no significant morphological changes versus control, 7.5 μM TAIII monotherapy demonstrated greater cytotoxicity than the combination therapy (5 μM gefitinib + 7.5 μM TAIII). These findings suggest that the combination regimen did not enhance therapeutic efficacy against H1975 (EGFR T790M) cells compared with TAIII monotherapy at the tested concentrations.

To investigate the molecular mechanisms underlying the drug interactions, we analyzed EGFR signaling pathway modulation following combination treatment using Western blotting. As demonstrated in Figure 6C, gefitinib monotherapy significantly increased p-ERK (Y204) phosphorylation compared with the control group, while total EGFR, AKT, and ERK levels remained unchanged—a pattern consistent with established resistance mechanisms [29]. In contrast, TAIII single-agent treatment markedly reduced both p-EGFR (Y1068) and p-ERK (Y204) levels. Notably, the combination therapy did not enhance this suppression beyond what was achieved with TAIII alone. These findings demonstrate that TAIII exerts its antiproliferative effects on H1975 cells primarily through inhibition of EGFR and its downstream ERK pathway. Conversely, gefitinib appears to promote resistance via ERK pathway activation. The absence of enhanced pathway suppression in the combination treatment explains the lack of synergistic effect observed between the two compounds.

### 2.7. In Vivo Efficacy of TAIII Against H1975 Xenografts

**Figure 7:**
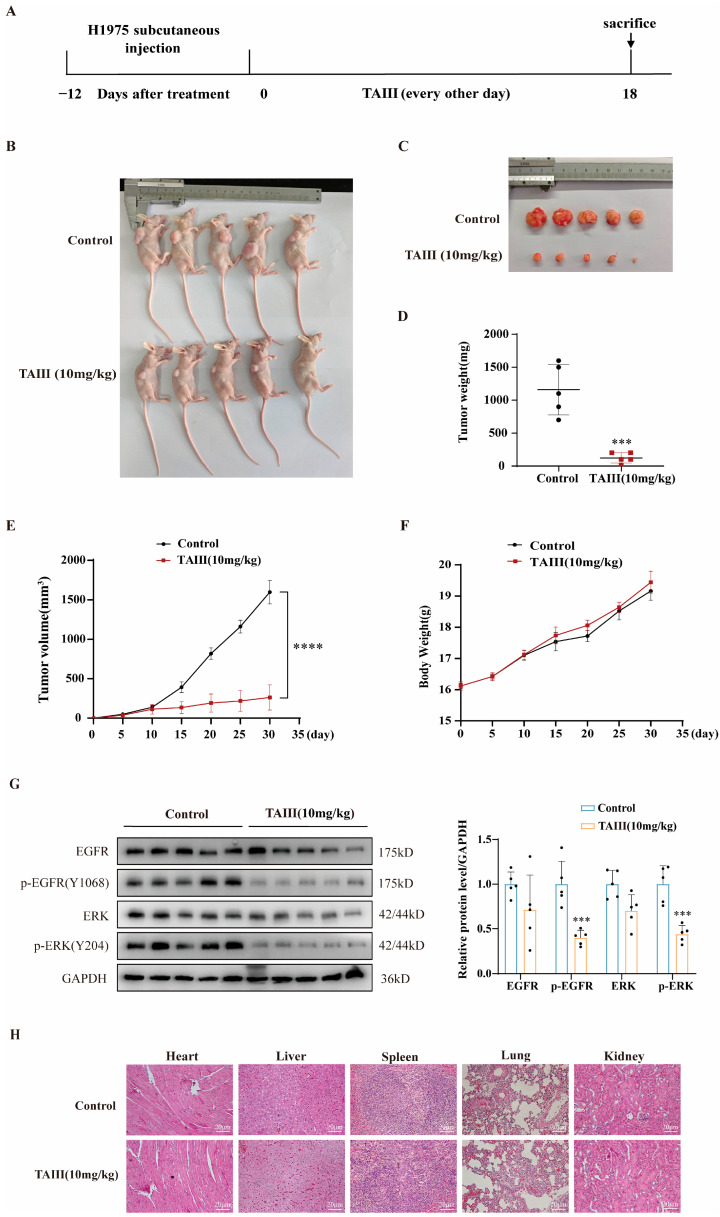
Studies on the inhibition of H1975-resistant strains by TAIII in vivo. In vivo antitumor efficacy of Timosaponin AIII in H1975 xenograft models. (**A**) Schematic illustration of the experimental design. (**B**,**C**) Representative images and quantitative analysis of tumor size from control and TAIII-treated groups at study endpoint. (**D**) Comparison of excised tumor weights between experimental groups. (**E**) Tumor growth kinetics monitored throughout the treatment period. (**F**) Body weight changes of mice during treatment as an indicator of treatment tolerability. (**G**) Western blotting analysis of phosphorylation status of EGFR and downstream signaling proteins in tumor tissues. (**H**) Heart, liver, spleen, lung, and kidney tissues from experimental animals were sectioned and stained with H&E. Scale bar: 20 μm. The data are presented as the mean ± SD (*n* = 5). Statistical significance was determined by unpaired *t*-tests. The symbols used to indicate significance levels are as follows: *** *p* < 0.001, **** *p* < 0.0001.

To assess the in vivo antitumor efficacy of TAIII against EGFR T790M-mutated H1975 cells, we established a subcutaneous xenograft model in BALB/c-nu nude mice. As illustrated in Figure 7A, H1975 cells were inoculated into the right dorsal flank of mice. Tumor progression and animal health were monitored through weekly measurements of tumor volume and body weight (Figure 7F). When tumors reached approximately 100 mm^3^ (day 12 post-implantation), mice were randomized into two treatment groups (*n* = 5/group) receiving either a vehicle control (PBS containing 2% DMSO and 1% Tween 80) or TAIII (10 mg/kg), administered every other day for 18 days. The treatment regimen continued until day 31, when mice were euthanized for terminal tumor analysis. Quantitative evaluation revealed significant antitumor activity of TAIII, as evidenced by reduced final tumor volumes (Figure 7B,C), decreased tumor weights (Figure 7D,E), and significant inhibition of tumor growth kinetics (*p* < 0.0001 vs. control). Notably, TAIII treatment did not induce significant body weight changes (Figure 7F), suggesting minimal systemic toxicity at the administered dose. These findings collectively demonstrate that TAIII effectively suppresses the growth of EGFR T790M-mutant H1975 xenografts in vivo.

To elucidate the molecular mechanism underlying the antitumor effects of TAIII on H1975 xenografts, we performed Western blotting analysis of key EGFR signaling pathway components. Quantitative assessment revealed significant downregulation of phosphorylated EGFR (Y1068) and ERK (Y204) in TAIII-treated tumors compared with vehicle controls (*p* < 0.05). While total EGFR and ERK protein levels showed a decreasing trend, these changes did not reach statistical significance (Figure 7G). These data collectively establish that TAIII exerts its antitumor activity in vivo primarily through the inhibition of EGFR phosphorylation and the consequent suppression of downstream ERK signaling.

To evaluate the systemic toxicity profile of TAIII, we conducted comprehensive histopathological analysis of major organs (heart, liver, spleen, lungs, and kidneys) using hematoxylin and eosin (H&E) staining. Histological examination revealed that no significant histopathological alterations were observed in the treatment group animals: cardiac tissue displayed regularly arranged myocardial fibers without inflammatory infiltration or fibrotic lesions; hepatic sections exhibited intact lobular architecture with morphologically normal hepatocytes and no portal tract abnormalities; splenic tissue showed clear demarcation between white and red pulp with homogeneous lymphocyte distribution; pulmonary tissue maintained normal alveolar structure without interstitial edema or inflammatory cell infiltration; renal sections demonstrated preserved glomerular morphology and tubular epithelium lacking degenerative or necrotic changes (Figure 7H), supporting the favorable safety profile of TAIII and providing crucial preclinical evidence for its therapeutic potential.

## 3. Discussion

Lung cancer remains the most lethal malignancy globally, ranking as the second most commonly diagnosed cancer and accounting for the highest number of cancer-related deaths worldwide [8]. The epidermal growth factor receptor (EGFR), a transmembrane tyrosine kinase receptor, serves as a critical regulator of cellular proliferation and survival [13]. Notably, EGFR is frequently overexpressed or mutated in various epithelial malignancies, making it one of the most clinically validated molecular targets for precision cancer therapy [37]. The development of EGFR tyrosine kinase inhibitors (EGFR-TKIs) has revolutionized targeted therapy for non-small-cell lung cancer (NSCLC) [38]. Gefitinib, as the first-generation EGFR-TKI, demonstrated promising initial clinical responses; however, its therapeutic efficacy is frequently limited by acquired resistance, predominantly mediated by the EGFR T790M secondary mutation [39]. While second-generation inhibitors (e.g., afatinib) were designed to address this resistance, their clinical utility is constrained by a narrow therapeutic index and dose-limiting toxicities [40]. Third-generation TKIs (e.g., osimertinib) effectively target T790M-mutant EGFR, yet their long-term efficacy is compromised by the emergence of tertiary mutations (e.g., C797S) and substantial treatment costs that limit patient accessibility [41]. These clinical challenges underscore the critical need for novel therapeutic approaches that can either prevent or overcome resistance to first-line EGFR-TKIs while maintaining favorable safety and cost-effectiveness profiles.

Therefore, this study investigated the pharmacodynamics and underlying mechanism of TAIII in H1975 (EGFR T790M) cells, both in vitro and in vivo. TAIII markedly suppressed the proliferation and migration of H1975 cells, prompting further mechanistic exploration. TAIII significantly attenuated the phosphorylation of EGFR and its downstream signaling proteins. Notably, the reduction in p-EGFR (Tyr1068) occurred prior to changes in other downstream effectors. Upon EGF-induced activation of the EGFR pathway, phosphorylation levels of EGFR and its downstream targets were substantially elevated, whereas TAIII treatment concentration-dependently reversed this effect. Consistent with these findings, EGF stimulation significantly enhanced H1975 cell proliferation and migration, whereas TAIII counteracted these effects. Furthermore, EGFR knockdown led to diminished phosphorylation of both EGFR and ERK, confirming that TAIII exerts its antiproliferative and antimigratory effects by suppressing EGFR-mediated signaling via phosphorylation inhibition.

The T790M mutation (replacement of Thr790 by Met) represents the primary mechanism of gefitinib resistance, accounting for 50–60% of resistance cases [42]. Structurally, the Met side chain is larger than the Thr side chain, occupying the entrance region of the ATP-binding pocket and blocking the entry path of gefitinib. Additionally, the hydrophobic side chain of Met increases the hydrophobicity of the binding pocket, disrupting the balance of the original hydrophobic interactions of gefitinib. In the wild-type EGFR, Thr790 may form weak hydrogen bonds with gefitinib via its hydroxyl group, whereas the Met790 mutation is unable to provide a hydrogen bond donor, resulting in a broken hydrogen bond network and weakening the binding strength of the drug to the target [43,44]. Given TAIII’s demonstrated efficacy in inhibiting H1975 (EGFR T790M) cells and suppressing EGFR phosphorylation, we further investigated its binding mode using molecular docking. The results revealed that the saponin moiety of TAIII stably occupies the hydrophobic region of the EGFR binding pocket, forming a hydrogen bond with THR-776. Notably, TAIII’s binding is independent of the Thr790 site, rendering it unaffected by the T790M mutation—unlike gefitinib. The glycan moiety extends into the protein’s hydrophilic surface, where terminal hydroxyl groups form stabilizing hydrogen bonds (e.g., with PRO-770), enhancing overall binding stability. This unique binding mode allows TAIII to maintain robust inhibitory activity against EGFR phosphorylation and downstream signaling, even in the context of T790M-mediated resistance. Further validation via cellular thermal shift assay (CETSA) and solvent-induced protein precipitation confirmed TAIII–EGFR interaction, demonstrating that TAIII binding reduces EGFR degradation under thermal and solvent stress.

Building upon our findings that TAIII induces degradation of EGFR and its downstream signaling proteins at high concentrations, we sought to elucidate the underlying molecular mechanism. Initial experiments using the proteasome inhibitor MG-132 failed to prevent EGFR pathway degradation, ruling out proteasome-mediated mechanisms. We subsequently demonstrated that TAIII induces autophagy, as evidenced by reduced p62 expression, increased LC3-II conversion, and enhanced autophagic flux. To further verify that autophagy is responsible for TAIII-mediated EGFR degradation, we targeted ATG5, a key gene essential for autophagosome formation [39]. ATG5 knockdown resulted in decreased LC3II accumulation and substantially restored protein levels of EGFR, AKT, and ERK, demonstrating that TAIII-induced EGFR degradation occurs through the autophagy–lysosomal pathway. Consistently, the autophagy inhibitor 3-MA also attenuated the TAIII-induced decrease in EGFR, AKT, and ERK protein levels, further supporting the involvement of autophagy-mediated degradation. Notably, while TAIII has been reported to trigger protective autophagy in A549 cells [27], our data reveal a distinct mechanism in H1975 cells: ATG5 knockdown reversed TAIII-mediated cell death, indicating that the induced autophagy is nonprotective in this T790M-mutant context. These findings highlight cell type-specific differences in TAIII’s regulation of autophagy, providing critical insights for future investigations. The efficacy of TAIII against T790M-mutant NSCLC, along with its unique mode of action, highlights the therapeutic promise of botanical compounds in comprehensive NSCLC treatment strategies [45]. Unlike other natural agents such as resveratrol [46], luteolin [47], or curcumin [48], which exert multi-target indirect modulation, TAIII operates through a distinct dual mechanism that overcomes EGFR T790M-mediated resistance. Specifically, TAIII directly binds to the EGFR kinase domain at THR-776/PRO-770, inhibiting its phosphorylation. At higher concentrations, it additionally promotes EGFR degradation via the autophagy–lysosome pathway. This combination of direct kinase inhibition and induced protein degradation represents a novel two-pronged strategy for suppressing EGFR signaling.

Certain herbal extracts exhibit potent antitumor activity by modulating multiple pathways involved in cancer cell survival and proliferation [49]. In addition, accumulating evidence suggests that specific phytochemicals derived from traditional Chinese medicine may enhance tumor sensitivity to chemotherapeutic agents while mitigating treatment-related adverse effects [50]. Gefitinib, a first-line tyrosine kinase inhibitor for advanced EGFR-mutant non-small-cell lung cancer (NSCLC), effectively suppresses EGFR-mediated oncogenic signaling [51]. However, approximately 50–60% of patients develop acquired resistance within 10 months of treatment, primarily through the T790M gatekeeper mutation [52]. Based on our previous findings demonstrating TAIII’s remarkable efficacy against gefitinib-resistant H1975 (EGFR T790M) cells, we systematically evaluated potential synergistic effects between TAIII and gefitinib through combination index (CI) analysis and proteomic profiling. Notably, our results revealed that TAIII monotherapy exhibited superior potency compared with combination treatment, with Western blotting analysis showing more pronounced suppression of p-EGFR (Tyr1068) and p-ERK levels. This phenomenon may be attributed to gefitinib-induced paradoxical activation of ERK1/2 in resistant cells through the MSI2-AGO2/miR-30a-3p-CGRRF1 regulatory axis, which subsequently activates the KRAS/ERK pathway to promote tumor progression and drug resistance [53].

Despite the promising antitumor activity of TAIII observed in preclinical studies—including in vitro assays and animal models of EGFR T790M-mutated NSCLC—several challenges must be addressed to advance its clinical translation. First, although direct binding between TAIII and EGFR has been confirmed through molecular docking, CETSA, and SIP assays, the potential for unidentified off-target effects cannot be fully excluded. Further validation using more comprehensive chemical biology approaches such as pull-down assays combined with mass spectrometry is warranted. Second, as a saponin compound, TAIII suffers from low oral bioavailability and poor metabolic stability, which are common limitations among saponin derivatives [54]. Pharmacokinetic studies in rats have shown that TAIII is rapidly eliminated, with an elimination half-life (t_1/2_) of 2.60 ± 0.56 h and a peak plasma concentration (Cmax) of 304.6 ± 66.8 ng/mL at a dose of 50 mg/kg [55]. These unfavorable pharmacokinetic properties underscore the need for advanced drug delivery strategies to enhance its bioavailability. Encouragingly, recent developments—such as anti-CD44 antibody-modified TAIII liposomes (CD44-LP) [56]—have markedly improved its pharmacokinetic profile, extending its half-life by 10.7–14.2-fold and achieving a tumor inhibition rate of 55.2% (7.2 times that of free TAIII), without significant systemic toxicity. Third, while TAIII has demonstrated a favorable safety profile in animal studies, these models often utilize immunodeficient mice that lack a humanized immune microenvironment. Future evaluations using humanized mouse models and patient-derived organoids will be essential to better predict clinical behavior. It is also important to note that although the multi-target nature of saponins may contribute to enhanced antitumor efficacy, it also introduces potential risks. Thus, comprehensive toxicological profiling and systematic pharmacological investigations will be critical in subsequent development stages.

## 4. Materials and Methods

### 4.1. Cell Culture

The human non-small-cell lung cancer cell lines A549 and H1975 and the normal alveolar epithelial cell line BEAS-2B were obtained from the Cell Bank of the Chinese Academy of Sciences (Shanghai, China) and Starfish (Suzhou, China), respectively. The cells were cultured in RPMI 1640 medium (Gibco, C11875500BT, Thermo Fisher Scientific, Waltham, MA, USA) supplemented with 10% fetal bovine serum (FBS, Gibco, 2800811, Thermo Fisher Scientific, Sao Paulo, Brazil) and 1% antibiotic solution (10,000 U/mL penicillin and 100 mg/mL streptomycin) at 37 °C in a 5% CO_2_ incubator.

### 4.2. Chemicals and Reagents

TAIII was purchased from Chengdu Pufide Biotechnology Co. (Chengdu, China, Cat #JOT-10101). The purity of the compound was measured using HPLC and was ≥98.69%. It was dissolved in DMSO (Sigma, St. Louis, MO, USA) to prepare a 10 mM stock solution. For in vitro experiments, the final concentrations of TAIII used were 0, 2.5, 5, and 10 mM. The percentage of DMSO in the control and treatment groups was 0.1% (*v*/*v*). Cycloheximide (CHX), MG132, and 3-methyladenine (3-MA) were obtained from MedChemExpress (Monmouth Junction, NJ, USA). The antibodies used for Western blotting were as follows: EGFR (v1010) (1:1000, Cat. #BS1533), PCNA (1:1000, Cat. #BS42666), Ki67 (1:1000, Cat. #BS40169), AKT (1:1000, Cat. #BS40169), p-AKT (Ser473) (1:1000, Cat. #BS43453), ERK (1:1000, Cat. #AP0485), STAT3 (1:1000, Cat. #BS45191), p-STAT3 (Tyr705) (1:1000, Cat. #BS43472), LC3I/II (1:1000, Cat. #BS7644), and GAPDH (1:10,000, Cat. #MB66349) from Bioworld (Bloomington, MN, USA); p-EGFR (Tyr1068) (1:1000, Cat. #11862) and p-ERK (1:2000, Cat. #4370) from Cell Signaling Technology (Danvers, MA, USA); and E-cadherin (1:1000, Cat. #20874-1-AP), N-cadherin (1:1000, Cat. #22018-1-AP), and β-actin (1:10,000, Cat. #66009-1-Ig) from Proteintech Group, Inc. (Chicago, IL, USA).

### 4.3. CCK8 Assay

The cells were seeded in 96-well plates at a density of 5 × 10^3^ cells per well and cultured overnight. Following drug treatment for the designated period, 10 μL of CCK8 reagent was added to each well. After 2–4 h, the absorbances of all the wells were measured at a wavelength of 450 nm via an enzyme marker (Vazyme, Nanjing, Jiangsu, China). The cell viability was calculated on the basis of absorbances.

### 4.4. EdU Staining Assay

An EdU-555 Cell Proliferation Assay Kit (Biyun Tian, Shanghai, China) was used to detect cell proliferation. Cells were inoculated in 24-well plates at 5 × 10^4^ cells per well and cultured overnight. After the treatment, prewarmed (37 °C) EdU working solution (10 μM) was added to each well, and cells were cultured for an additional 2 h. The cells were then fixed with 4% paraformaldehyde for 10–15 min and permeabilized with 0.3% Triton X-100 for 10–15 min at room temperature. After three PBS washes, the Click reaction solution was added, followed by nuclear counterstaining with DAPI (1 μg/mL) for 10 min. Fluorescence imaging was conducted using an inverted fluorescence microscope (Olympus, Tokyo, Japan). Quantification of EdU-positive cells was performed using ImageJ software (version 1.53t; National Institutes of Health, Bethesda, MD, USA).

### 4.5. Transwell Migration Assay

Transwell chambers (pore size 8 μm; Corning, New York, NY, USA) were inserted into 24-well culture plates. The lower chamber was filled with 500 μL of complete culture medium supplemented with 15% fetal bovine serum (FBS) as a chemoattractant. Cells were dissociated with trypsin and resuspended in serum-free medium at a concentration of 5 × 10^5^ cells/mL. Subsequently, a 100 μL cell suspension containing 5 × 10^4^ cells was added to each upper chamber. The chambers were incubated at 37 °C in a 5% CO_2_ incubator for 24 h. After incubation, the cells were fixed with methanol for 30 min, washed twice with PBS, and stained with 0.1% crystal violet for 15 min. Images were captured using an inverted microscope (Olympus, Tokyo, Japan). The number of cells was quantified using ImageJ software (NIH, USA).

### 4.6. Scratch Assay

Cells were trypsinized and seeded into 6-well plates at a density of 5 × 10^5^ cells/well in complete growth medium and incubated for 24 h. A uniform wound was created in each monolayer using a sterile 200 μL pipette tip (Axygen, Union City, CA, USA). The wells were washed gently with PBS to remove detached cells. Then, low-serum medium (containing 2% FBS) was added to each well. Images were captured using an inverted microscope (Olympus, Tokyo, Japan) as a 0 h control. Subsequent images were acquired at 24-h intervals under identical conditions. Wound areas were quantified using ImageJ software (NIH, USA).

### 4.7. Molecular Docking

The I-TASSER tool (https://zhanglab.ccmb.med.umich.edu/I-TASSER/, accessed on 10 January 2024) was employed for structural modeling of the EGFR protein, whereas the PubChem database (https://pubchem.ncbi.nlm.nih.gov/, accessed on 10 January 2024) was utilized for structural modeling of TAIII. Molecular docking analysis was conducted via Autodock Tools 1.5.6 software (developed by the National Institutes of Health, Bethesda, MD, USA).

### 4.8. Cellular Thermal Shift Assay (CETSA)

H1975 cells were cultured in 10 mm dishes until reaching >90% confluency. The cells were lysed by RIPA lysis solution, followed by centrifugation at 4 °C and 12,000× *g* for 15 min to collect the supernatant. The supernatants were divided into a control group (DMSO) and a treatment group (TAIII, 20 μM). Following 1 h compound incubation at 4 °C, samples were subjected to thermal denaturation at various temperatures (50, 54, 58, 62, 66, 70, and 74 °C) for 3 min using a thermal cycler, and then immediately cooled on ice. Thermally denatured proteins were removed by secondary centrifugation (12,000× *g*, 15 min, 4 °C). Western blotting analysis was used to detect the target protein.

### 4.9. Solvent-Induced Protein Precipitation (SIP)

H1975 cells were cultured in 10 mm dishes until reaching >90% confluency. The cells were lysed by RIPA lysis solution, followed by centrifugation at 4 °C and 12,000× *g* for 15 min to collect the supernatant. The supernatants were divided into a control group (DMSO) and a treatment group (TAIII, 20 μM). Following 1 h incubation with gentle agitation (200 rpm) at room temperature, each group was subdivided into six 80 μL aliquots. A precipitation cocktail (acetone/ethanol/acetic acid = 50:50:0.1, *v*/*v*/*v*) was titrated into each aliquot at final concentrations of 9%, 11%, 13%, 15%, 17%, and 19% (*v*/*v*). Samples were equilibrated (720× *g*, 20 min, 4 °C) to allow protein precipitation, followed by pelleting of insoluble aggregates (12,000× *g*, 15 min, 4 °C). Finally, the expression of EGFR was analyzed via Western blotting.

### 4.10. Quantitative Reverse Transcription-PCR (qRT-PCR)

Total RNA was extracted with TRIzol reagent (Invitrogen, Carlsbad, CA, USA) and reverse transcribed into cDNA using HiScript III RT SuperMix (R323-01, Vazyme, Nanjing, China). qPCR was performed using AceQ qPCR SYBR Green Master Mix (Q111-02/03, Vazyme, Nanjing, China) on an Applied Biosystems thermal cycler with the following protocol: 95 °C for 15 min, 95 °C for 10 s, 60 °C for 30 s, and 40 cycles. Melt curve analysis was performed to confirm amplification specificity. Relative gene expression was quantified using the 2^−ΔΔCT^ method, with GAPDH serving as the endogenous control. Gene-specific primers were designed based on sequences retrieved from the NCBI database (Bethesda, MD, USA).

The primer sequences used were EGFR (F: 5′-AGGCACGAGTAACAAGCTCAC-3′ R: 5′-ATGAGGACATAACCAGCCACC-3′), GAPDH (F: 5′-GGAGCGAGATCCCTCCAAAAT-3′; R: 5′-GGCTGTTGTCATACTTCTCATGG-3′).

### 4.11. Transfection

Cells were seeded into culture dishes at a density optimized to achieve 50–60% confluency at the time of transfection. The siRNA mixture was then mixed with GenJet (SignaGen, SL100489, Frederick, MD, USA) and incubated for 15 min at room temperature. The siRNA–GenJet complexes were then added dropwise to the culture medium, followed by gentle agitation to ensure uniform distribution. The cells were subsequently cultured at 37 °C with 5% CO_2_. The medium was replaced with serum-containing complete medium at 6 h post-transfection. The transfection efficiency was evaluated 48 h after transfection using Western blotting assay.

### 4.12. GFP-RFP-LC3 Dual Fluorescent Labeling Assay

The pGFP-RFP-LC3 plasmid was kindly provided by Professor Xiaoping Wang from China Pharmaceutical University. The LC3 coding sequence was amplified by PCR and cloned into the pCMV-GFP-RFP vector using XhoI and SalI restriction sites. H1975 cells were transfected with the constructed pGFP-RFP-LC3 plasmid. After 24 h, transfected cells were treated with 10 μM TAIII for 12 h, followed by fixation with 4% paraformaldehyde. Fluorescence imaging was performed using an Olympus inverted fluorescence microscope. In this experiment, yellow fluorescent puncta were formed by the merging of GFP green fluorescent signals and RFP red fluorescent signals.

### 4.13. Western Blotting

Proteins were extracted by RIPA buffer supplemented with protease and phosphatase inhibitors (Thermo Fisher Scientific, Waltham, MA, USA), and the protein concentration was determined via the BCA assay (Pierce™, Thermo Fisher Scientific, Waltham, MA, USA). The samples were resolved by SDS-PAGE on 8%, 10%, or 15% polyacrylamide gels under reducing conditions. Proteins were then electrophoretically transferred onto 0.45 μm PVDF membranes (Millipore, Burlington, MA, USA) using a wet transfer system. The membranes were blocked with 5% nonfat milk for 1.5 h, and then incubated with primary antibody overnight at 4 °C. After washing, membranes were probed with HRP-conjugated secondary antibodies (1:5000) for 1 h at room temperature. Protein bands were visualized using an enhanced chemiluminescence (ECL) reagent (Thermo Fisher Scientific, Waltham, MA, USA) and imaged on a Clinx ChemiScope imaging system (Qinxiang, Shanghai, China). Band intensities were quantified using ImageJ (NIH) or similar software.

### 4.14. H&E Staining

Tissue samples were fixed in 4% paraformaldehyde for 24–48 h at 4 °C, followed by dehydration through a graded ethanol series (70%, 80%, 90%, and 100%), cleared in xylene, and embedded in paraffin. Sections of 4–5 μm thickness were cut using a microtome, mounted on glass slides, and dried at 60 °C for 1 h. After deparaffinization in xylene and rehydration through descending alcohol concentrations (100%, 95%, and 70%), slides were stained with hematoxylin for 5–8 min, rinsed in running tap water, differentiated in 1% acid alcohol for 30 s, and blued in 0.2% ammonia water. Counterstaining was performed with eosin for 1–2 min, followed by dehydration through ascending alcohols, clearing in xylene, and mounting with neutral balsam. Microscopic examination was conducted using a light microscope, with representative images captured using a digital camera system. All staining procedures included appropriate positive and negative controls, and at least three independent tissue sections were analyzed per sample to ensure reproducibility.

### 4.15. Tumor Xenograft

Female athymic BALB/c nu/nu mice (6 weeks, 14–16 g) were purchased from Gempharmatech Co., Ltd. (Nanjing, China). H1975 cells (3 × 10^6^ cells in 100 μL PBS) were subcutaneously injected into each mouse. When the average tumor volume reached approximately 100 mm^3^, the mice were randomly divided into two groups (*n* = 5 per group): a control group and a TAIII treatment group. TAIII was administered via intraperitoneal injection at a dose of 10 mg/kg, with an injection volume of 10 mL/kg, every other day for a total of 9 doses. The control group received an equal volume of PBS containing 2% DMSO and 1% Tween 80 via intraperitoneal injection. Tumor volumes (calculated as V = 0.5 × length × width^2^) and body weights were measured twice weekly. Mice were sacrificed 6 h after the final injection. Tumors were excised, weighed, and snap-frozen for further analysis. Major organs (heart, liver, kidneys, spleen) were collected for histological and Western blotting analysis. All animal procedures were conducted in accordance with the ethical guidelines of the Animal Ethical and Welfare Committee of Nanjing Medical University (Approval No. 2306008-1).

### 4.16. Statistical Analysis

Statistical analysis for significance was performed using GraphPad Prism 9.0 (GraphPad Software, San Diego, CA, USA). Unpaired *t*-tests were used for comparisons between two independent groups. One-way ANOVA followed by Tukey’s multiple comparisons test was used for comparisons among three or more groups. One-way repeated measures ANOVA with Bonferroni’s post hoc test was applied for repeated measurements over time within the same group. Two-way ANOVA with Bonferroni’s post hoc test was used to assess the interaction effects of two factors. The significance level was set at *p* < 0.05. All experiments were independently repeated at least three times.

## 5. Conclusions

Our research systematically evaluated the therapeutic potential of TAIII against EGFR T790M (NSCLC) via in vitro and in vivo experiments. The results showed that TAIII had a significant inhibitory effect on the growth of EGFR T790M NSCLC and could effectively alleviate the drug resistance caused by the EGFR T790M mutation. Our findings not only lay a solid theoretical foundation for the clinical application of TAIII but also represent a significant step forward in the ongoing efforts to address the challenge of drug resistance in EGFR-mutated NSCLC. These results underscore the importance of exploring natural compounds as a source of novel anticancer agents and highlight the potential of TAIII to drive further advancements in the field of lung cancer treatment. Future research will focus on optimizing the pharmacokinetic properties of TAIII and exploring its potential synergistic effects with other targeted therapies to maximize its clinical impact.

## Data Availability

The raw datasets utilized and analyzed in this study are included in the article. Further inquiries can be directed to the corresponding author.

## Abbreviations

The following abbreviations are used in this manuscript:

TAIII	Timosaponin AIII
NSCLC	Non-small-cell lung cancer
EGFR	Epidermal growth factor receptor
EGF	Epidermal growth factor
ATG5	Autophagy-related gene 5
DMSO	Dimethyl sulfoxide
PCNA	Proliferating cell nuclear antigen
Ki67	Marker of proliferation
Bcl2	B-cell lymphoma-2
ERK	Extracellular signal-regulated kinase
AKT	Protein Kinase B
CCK8	Cell Counting Kit8
BCA	Bicinchoninic Acid
PCR	Polymerase Chain Reaction
RT-PCR	Reverse Transcription-Polymerase Chain Reaction
WB	Western blot
FBS	Fetal bovine serum
PBS	Phosphate buffer saline
APS	Ammonium acid
DAPI	4′,6-Diamidino-2′-phenylindole
CQ	Chloroquine
EdU	5-Ethynyl-2′-deoxyuridine
ECL	Enhanced chemiluminescence
EDTA	Ethylene diamine tetraacetic acid
DEPC	Diethyl pyrocarbonate
CHX	Cycloheximide
MG132	Z-Leu-Leu-Leu-al
ACTB	Beta-actin
GAPDH	Glyceraldehyde-3-phosphate dehydrogenase
